# Vaccination with Recombinant Baculovirus Expressing Ranavirus Major Capsid Protein Induces Protective Immunity in Chinese Giant Salamander, *Andrias davidianus*

**DOI:** 10.3390/v9080195

**Published:** 2017-07-25

**Authors:** Xiaoyuan Zhou, Xinglang Zhang, Yahui Han, Qiuhong Jia, Hongwei Gao

**Affiliations:** Yellow River Fisheries Research Institute, Chinese Academy of Fishery Sciences, 2 Fenghui Road, South of Sanqiao, District of Weiyang, Xi’an 710086, China; zxlang029@sina.com (X.Z.); hanyahui-2006@163.com (Y.H.); jiaxiu_001@163.com (Q.J.); gaohongwei8001@126.com (H.G.)

**Keywords:** Chinese giant salamander, *Ranavirus*, major capsid protein, vaccination, protective immunity

## Abstract

The Chinese giant salamander iridovirus (CGSIV), belonging to the genus *Ranavirus* in the family *Iridoviridae*, is the causative agent of an emerging infectious disease causing high mortality of more than 90% and economic losses in Chinese giant salamanders in China. In this study, a recombinant baculovirus-based vaccine expressing the CGSIV major capsid protein (MCP) was developed and its protective immunity in Chinese giant salamanders was evaluated. The recombinant *Autographa californica* nucleopolyhedrosis virus (AcNPV), expressing CGSIV MCP, designated as AcNPV-MCP, was generated with the highest titers of 1 × 10^8^ plaque forming units/mL (PFU/mL) and confirmed by Western blot and indirect immunofluorescence (IIF) assays. Western blot analysis revealed that the expressed MCP reacted with mouse anti-MCP monoclonal antibodies at the band of about 53 kDa. The results of IIF indicated that the MCP was expressed in the infected *Spodoptera frugiperda* 9 (Sf9) cells with the recombinant baculovirus, and the Chinese giant salamander muscle cells also transduced with the AcNPV-MCP. Immunization with the recombinant baculovirus of AcNPV-MCP elicited robust specific humoral immune responses detected by ELISA and neutralization assays and potent cellular immune responses in Chinese giant salamanders. Importantly, the effective immunization conferred highly protective immunity for Chinese giant salamanders against CGSIV challenge and produced a relative percent of survival rate of 84%. Thus, the recombinant baculovirus expressing CGSIV MCP can induce significant immune responses involving both humoral and cell-mediated immunity in Chinese giant salamanders and might represent a potential baculovirus based vaccine candidate for Chinese giant salamanders against CGSIV.

## 1. Introduction

Members of the genus *Ranavirus* in the family *Iridoviridae* are well known for causing increasingly reported declines and die-offs of amphibians around the world and have been recognized as threats to global amphibian populations and biodiversity [1]. More recently, a ranavirus was isolated and identified from diseased Chinese giant salamanders (*Andrias davidianus*) in China and designated as Chinese giant salamander iridovirus (CGSIV) [2,3,4], which is the causative agent of an emerging infectious disease causing high mortality of more than 90% in farmed Chinese giant salamanders and resulting in great economic losses to the breeding and cultivation industry of Chinese giant salamanders [5,6]. Clinically, the syndrome caused by this disease is characterized by skin ulceration, toe necrosis, cephalic swellings, external hemorrhages, gray-black liver, and friable lesions of the kidneys and spleens [4,5].

Unlike bacterial diseases, chemicals and antibiotics have little effective therapy for viral infections including CGSIV. Vaccination has been proved to be a potent tool for effectively controlling viral diseases. As the preferred vaccine type, the inactivated whole-virus vaccine could raise high safety concerns, although its protective effectiveness against CGSIV in Chinese giant salamanders has been established [7]. Alternative methods, recombinant subunit vaccine and DNA vaccine based on the major capsid protein (MCP) or the gene encoding MCP (a well-known immunogenic protein of the ranavirus [8,9]), have been developed as vaccine candidates for Chinese giant salamanders [10,11]. For the former MCP-based subunit vaccine prepared by use of *Picha pastoris* yeast system, several problems, such as over-glycosylation and methanol residue, still need to be solved [12]. Moreover, the protective efficacy of this vaccine is less than satisfactory [13]. Thus, it is imperative to investigate safer and more efficient CGSIV vaccine by use of novel vectors.

The baculovirus *Autographa californica* nucleopolyhedrosis virus (AcNPV), a double-stranded DNA virus that naturally only infects insects [14], has proven to be an efficient vaccine vector with several attractive advantages such as its good bio-safety, the large-capacity of foreign genes, and better post-translational modification [15]. Currently, the baculovirus system has been used to successfully express many vaccine candidate antigens with near-native forms, which induced both humoral and cell mediated immunity against viral infections [16,17,18]. Therefore, in this study we aim to develop a recombinant baculovirus based vaccine expressing CGSIV MCP and evaluate its potential as a vaccine candidate against CGSIV infection in Chinese giant salamanders.

## 2. Materials and Methods

### 2.1. Ethics Statement

Animal handling was conducted according to the recommendations in the Regulations for the Administration of Affairs Concerning Experimental Animals of PR China. The protocol was approved by the Animal Care and Use Committee of Yellow River Fisheries Research Institute, Chinese Academy of Fishery Sciences (CAS-YRFRI-2017-003), and all efforts were made to minimize the suffering of the animals.

### 2.2. Plasmid, Viruses, Cells and Animals

Plasmid pMD-18T-MCP harboring the full-length gene of CGSIV MCP (Gen-Bank accession no. K023635) was previously prepared by the laboratory. CGSIV-LY strain (CGSIV/LY/2012/12) was originally isolated and identified by the laboratory, which was grown in epithelioma papilloma cyprini (EPC) cells (China Center for Type Culture collection, Wuhan University, Wuhan, China) at 25 °C in Dulbecco’s modified Eagle’s medium (DMEM) supplemented with 10% fetal bovine serum (FBS) in culture medium and whose titer was determined as described previously [19]. *Spodoptera frugiperda* 9 (Sf9) cells were grown and maintained at 27 °C in Sf-900 II serum-free medium (SFM) (Invitrogen, San Diego, CA, USA). Chinese giant salamander muscle (GSM) cells were maintained at 20 °C in TC199 medium (Sigma, St. Louis, Mo, USA) containing 100 units mL^−1^ penicillin (Sigma) and 100 μg/mL streptomycin (Sigma) and 10% (*v*/*v*) heat-inactivated fetal calf serum (FCS). Healthy Chinese giant salamanders (about three years old and weighing 1.0 ± 0.25 kg) that were confirmed to be free of CGSIV by PCR were obtained from a farm in Liuba County, Shaanxi Province, PR China. These animals were kept in cement tanks sized 1.40 m × 1.0 m × 0.60 m at 18–20 °C and fed daily with diced salmon meat for two weeks before the experiment was initiated.

### 2.3. Generation of Recombinant Baculovirus

PCR was performed with pMD-18T-MCP as the template by using MCP specific primers with introduced *Eco*R I and *Hin*d III sites (Table 1). Then, the purified PCR product of MCP was inserted into the commercial vector pFastBac1 (pFB1—Invitrogen, Thermo Fisher Scientific, Waltham, MA, USA) under the polyhedrin promoter to construct the recombinant plasmid of pFastBac-MCP. The plasmid DNA of pFastBac-MCP was then transformed into *Escherichia coli* DH10Bac chemically competent cells (Invitrogen) to generate a recombinant bacmid through site-specific transposition. The recombinant bacmid was confirmed as rBacmid-MCP by PCR using the M13 Forward (−40) and M13 Reverse primers together with the gene-specific primers. The recombinant bacmid DNA was transfected into Sf9 cells using Insect GeneJuice Transfection Reagent (Merk Millipore, MerkkGaA group, Darmstadt, Germany) according to the manufacturer’s protocol to generate the recombinant baculovirus. The supernatant of three days post-transfection (dpt) Sf9 cells containing the recombinant viruses was harvested as the first passage viral stock, which were amplified further by propagation in Sf9 cells with a multiplicity of infection (MOI) of 1 and assayed as described previously [20]. Over two consecutive passages, the resulting recombinant viruses with the highest titers of 1 × 10^8^ plaque forming units/mL (PFU/mL) were obtained in the supernatant and designated as AcNPV-MCP.

### 2.4. Expression and Purification of Major Capsid Protein

Sf9 cells (1 × 10^6^ cells/mL) were infected with the recombinant baculovirus AcNPV-MCP at a MOI of 5 and incubated at 27 °C for three days. After the cells were harvested, cell extracts were prepared as described previously [21] and then separated by a 12% reducing sodium dodecyl sulfate-polyacrylamide gel electrophoresis (SDS-PAGE). Then the harvested Sf9 cells were resuspended in lysis buffer, incubated on ice for 10 min, and then sonicated followed by centrifugation at 13,000 rpm for 30 min at 4 °C. MCP was purified from the resultant supernatant with immunomagnetic beads prepared by the mixture of 500 µL Beaver Beads Mag NHS (Beaverbio, Suzhou, China) with 350 µL of mouse anti-MCP monoclonal antibodies (MCP-mAb) prepared by the laboratory [22] according to the manufacturer’s instructions. The purity of the protein was checked by a 12% reducing SDS–PAGE, and the protein yield was determined according to the method of Bradford with the Bio-Rad Protein Assay kit (Bio-Rad, Hercules, CA, USA) using bovine serum albumin (Sigma, St. Louis, Mo, USA) as the standard. The highly purified MCP was used as an enzyme-linked immunosorbent assay (ELISA) coated antigen.

### 2.5. Western Blot

Protein extracts from baculovirus-infected Sf9 cells and the purified MCP were subjected to 12% reducing SDS–PAGE, then transferred to a polyviny lidene fluoride membrane (Bio-Rad) and probed with MCP-mAb primary antibodies and horseradish peroxidase (HRP) conjugated anti-rabbit IgG secondary antibody (Sigma, St. Louis, Mo, USA) respectively. Finally, the membrane was incubated with enhanced chemiluminescence (ECL) reagent (Thermo Fisher Scientific), and immunoreactive bands were then visualized under gel imaging (GeneGenius, Syngene, Cambridge, UK).

### 2.6. Indirect Immunofluorescence Assay (IIF)

Sf9 cells were seeded at a density of 2 × 10^6^ cells/well on sterile round cover slides in a six-well plate (Costar, Corning, NY, USA) and infected with recombinant baculoviruses at a MOI of 5 at 27 °C for 72 h. Following fixation with 4% paraformaldehyde overnight at 4 °C, the cells were incubated with MCP-mAb and then with fluorescein isothiocyanate (FITC)-conjugated anti-mouse IgG (Sigma-Aldrich). For nuclear staining, the slides were covered with a drop of Vectashield with Hoechest 33258 (Vector Laboratories, Burlingame, CA, USA). Bound antibodies were detected by fluorescence microscopy (BX-51, Olympus, Tokyo, Japan). GSM cells were seeded at a density of 5 × 10^5^ cells/well in another six-well plate and transduced with the recombinant baculoviruses at a MOI of 10 by a 1-h incubation, facilitating viral adsorption. After washing and the addition of fresh medium, the cells were incubated at 20 °C for 48 h incubation and fixation as above, the cells were incubated with MCP-mAb and then with cy3-labelled IgG (Biosource International, Camarillo, CA, USA). For nuclear staining, the slides were covered with a drop of Vectashield with DAPI (4,6-diamidino-2-phenylindole) (Vector Laboratories). Bound antibodies were detected as before.

### 2.7. Animals Immunization and Serum Samples Collection

Ninety Chinese giant salamanders were randomly divided into three groups (30 animals/group). Animals were immunized intramuscularly two times at three-week intervals with 1 × 10^8^ PFU of AcNPV-MCP, wild-type AcNPV (AcNPV-WT) and 1 mL phosphate buffered solution (PBS) respectively, designated as AcNPV-MCP group, AcNPV-WT group, and PBS control group. Sera from three animals per group were collected by tail bleeding at days 0, 7, 14, 21, 28, 35, and 42 after the primary immunization.

### 2.8. Indirect ELISA

MCP-specific antibodies present in serum samples from animals of three groups above were quantified by indirect ELISA. Precoated ELISA plates with the purified MCP with a final concentration of 2 µg/mL diluted in 50 mM NaHCO_3_ (pH 9.6) were incubated with serial dilutions of serum samples. Specific IgY antibodies were detected by using rabbit antiserum against Chinese giant salamander IgY prepared by the laboratory [23] and mouse-anti-rabbit-IgG-HRP conjugate (Sigma-Aldrich) as primary and secondary antibodies, respectively. The plates were developed with a peroxidase substrate-ate solution (3,3′,5,5′-tetramethylbenzidine). The optical density at 450 nm of each well was determined using a microplate reader (Bio-Rad). The mean absorbance value in duplicate wells was recorded, and the endpoint titers were expressed as the reciprocal of the highest sample dilution for which the optical density was equal to or greater than the mean optical density of preimmune sera.

### 2.9. Neutralization Assay

Serum samples were heat-inactivated for 30 min at 56 °C. Neutralization antibodies present in serum samples were detected as described previously [10]. Briefly, two-fold serial dilutions of serum (50 µL) in DMEM were mixed with an equal volume of DMEM containing 200 tissue culture infective dosage (TCID_50_) of CGSIV and the mixture was incubated at 25 °C for 1 h. Then the mixture was added in wells (100 µL per well) of a 96-well microplate containing EPC cell monolayers, followed by incubation at 25 °C for 1 h. The mixtures were gently aspirated and 200 µL of fresh DMEM supplemented with 2% FBS were added back to each well. Endpoint titers were determined after 5–7 days incubation at 25 °C and expressed as the reciprocal of the highest serum dilution resulting in neutralization of virus activity by 50%.

### 2.10. Splenocyte Proliferation

At two weeks after the booster immunization as described in this section, three animals for each group were sacrificed in MS 222(3-Aminobenzoic acid ethyl ester methanesulfonate) for spleen removal. After being isolated as described previously [24], the splenocytes (5 × 10^5^ cells/well) were plated in 96-well culture plates and cultured in 100 µL TC199 medium (Sigma) supplemented with 10% FBS at 20 °C. After 2 h inoculation, the splenocytes were stimulated with100 µL/well of the recombinant baculovirus AcNPV-MCP (experimental group) and 5 µL/well lipopolysaccharide (LPS) (1 mg/mL, Sigma, positive control) or 100 µL/well TC199 medium (negative control) at 20 °C for 62 h. Following the incubation with 10 µL/well of cell counting kit-8 (CCK-8) solution (Dojindo, Kumamoto, Japan) for 4 h, viability was assessed by measuring the optical density (OD) at 450 nm. The splenocyte proliferation was measured by the stimulation index (*SI*) calculated according to Equation (1):

(1) *SI* = (experimentor LPS OD_450_ − negative control OD_450_)/negative control OD_450_.

### 2.11. Quantitative Real-Time PCR

After the spleens of three animals from each group were sampled as described above, the total RNA was isolated from the spleens using Trizol Reagent (Invitrogen) according to the manufacturer’s instructions. Five micrograms of total RNA were treated with DNase I (Promega, Carlsbad, CA, USA) to remove any genomic DNA traces that might interfere with the PCR reactions and then used to obtain cDNA using the ReverTra Ace-a-Kit (Toyobo, Osaka, Japan). Quantitative real-time PCR was performed with SYBR green real-time PCR master mix reagents kit (Toyobo) by Applied Biosystems 7500 Real-Time PCR Systems (ABI, Thermo Fisher Scientific). The immune-related genes including type I interferon (*IFN*), interleukin-1β (*IL-1β*), tumor necrosis factor alpha (*TNF-α*), and toll-like receptor 9 (*TLR9*) were examined. Briefly, the real-time PCR assays were carried out with the specific primers of the selected genes (Table 1). The PCR reaction procedure was as follows: 95 °C for 5 min followed by 40 cycles of 95 °C for 15 s, 60 °C for 1 min, and 72 °C for 30 s. All samples were assayed in triplicate and *β-actin* gene was used as an endogenous control to normalize targets. The relative expression levels of the target genes were calculated with the 2^−∆∆*C*t^ method [25].

### 2.12. Challenge Test

Two weeks after the booster immunization, each animal of the three groups was challenged by inoculation intraperitoneally (ip) with 100 µL of live CGSIV (1.0 × 10^5.5^ TCID_50_/mL). The mortality was recorded daily for up to 25 days post-challenge. The relative percent survival (*RPS*) was calculated according to Amend [26] as in Equation (2):

(2) $$RPS=\left(1-\frac{\mathrm{mortality}\;\mathrm{in}\;\mathrm{vaccinated}\;\mathrm{animals}}{\mathrm{mortality}\;\mathrm{in}\;\mathrm{control}\;\mathrm{animals}}\right)\times 100\%.$$

### 2.13. Statistical Analysis

Data were presented as the mean ± standard deviation (SD) and all statistical analyses were done using SPSS 17.0 package (SPSS, Chicago, IL, USA). The Student *t*-tests were used to compare the differences in antibody response, SI index, and transcription levels of the immunized group with the PBS control group. If significant, the least significant difference test was further used. In all cases, differences were considered statistically significant at *p* < 0.05 or highly significant at *p* < 0.01.

## 3. Results

### 3.1. Generation of Recombinant Baculovirus Expressing CGSIV MCP

The gene of the MCP from CGSIV-LY strain was amplified by PCR using the plasmid of pMD-18T-MCP as the template and sub-cloned into pFastBac1 vector, generating pFastBac-MCP which was then transformed into *E. coli* DH10Bac, and the recombinant rBacmid-MCP was obtained after identification by PCR and DNA sequencing (data not shown). After transfection of Sf9 cells with the recombinant bacmid and further amplification, the recombinant baculovirus with the highest titer of 1 × 10^8^ PFU/mL was generated.

**Figure 1:**
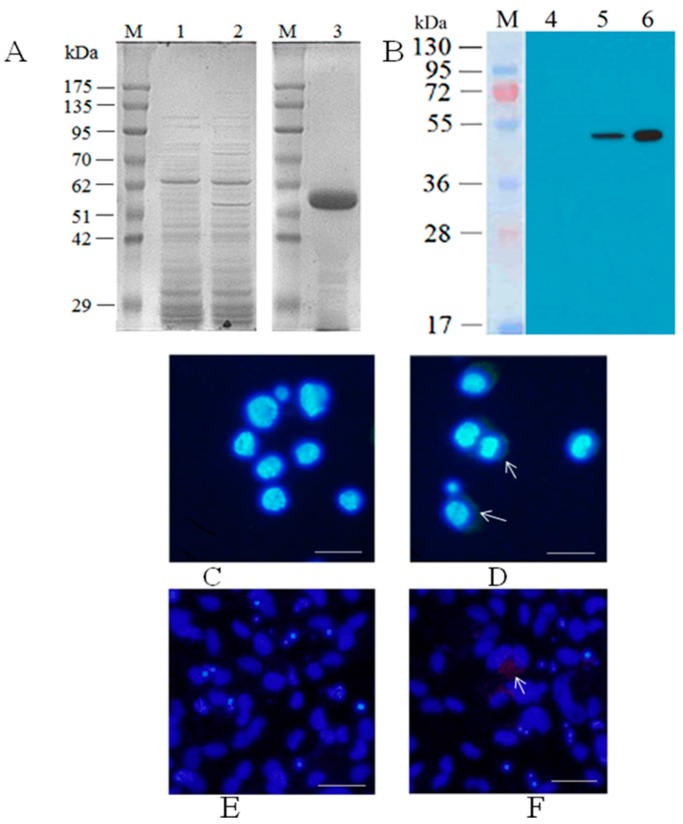
Expression of the major capsid protein (MCP). (**A**) Sodium dodecyl sulfate-polyacrylamide gel electrophoresis (SDS-PAGE) analysis, lane 1: normal *Spodoptera frugiperda* 9 (Sf9) cells (negative control); lane 2: Sf9 cells infected with the recombinant baculoviruses; lane 3: the purified MCP. (**B**) Western blot analysis, lane 1: normal Sf9 cells (negative control); lane 2: Sf9 cells infected with the recombinant baculoviruses; lane 3: the purified MCP. Primary monoclonal antibody is the mAb-MCP and secondary antibody is horseradish peroxidase conjugated goat anti-mouse IgG. (**C**,**D**) Expression analysis of MCP by infecting Sf9 cells with the recombinant baculoviruses *Autographa californica* nucleopolyhedrosis virus (AcNPV) expressing CGSIV MCP (AcNPV-MCP). At 72 h post-infection, Sf9 cells were fixed by 4% paraformaldehyde and analyzed by IIF using the mAb-MCP as primary antibody and fluorescein isothiocyanate-conjugated rabbit anti-mouse IgG as secondary antibody respectively. MCP-specific green fluorescence can be observed in the infected Sf9 cells (**D**) (indicated by arrows), whereas there was no specific fluorescence signal in the normal Sf9 cells (**C**). (**E**,**F**) Expression analysis of MCP by transducing the recombinant AcNPV-MCP in Chinese giant salamander muscle cells. The cells were incubated with MCP-mAb and then with cy3-labelled IgG, the nuclear was staining with DAPI. MCP-specific red fluorescence can be observed in the GSM cells transduced with the recombinant baculoviruses (indicated by arrows) (**F**), while no specific fluorescence signal was detected in the control (**E**). Scale bars indicate 40 µm.

To detect correct expression of MCP in the recombinant baculovirus, protein extracts from Sf9 cells infected with the recombinant baculovirus and the samples of the purified MCP were analyzed by SDS-PAGE. As shown in Figure 1A, a protein band corresponding to about 53 kDa was detected in both Sf9 cells infected with the recombinant baculovirus and the purified MCP with the prepared immunomagnetic beads binding MCP-mAb. Moreover, SDS-PAGE analysis of the purified MCP revealed that the MCP was highly purified, with a purity of over 95%, and the concentration of the purified MCP was approximately 1.05 mg/mL in accordance with the result of the Bradford protein assay (data not showed). The results of western blot assay indicated that MCP-mAb can recognize MCP expressed in the recombinant baculovirus with the same relative molecular mass of about 53 kDa. No specific band reactive with the MCP-mAb was observed in normal Sf9 cells. In addition, SDS-PAGE analysis of the purified MCP with the prepared immunomagnetic beads binding MCP-mAb revealed that the MCP was highly purified with a purity of over 95% (Figure 1B) and the concentration of the purified MCP was approximately 1.05 mg/mL in accordance with the result of the Bradford protein assay.

Additionally, we performed IIF with MCP-mAb as the primary antibody to confirm the expression of MCP in Sf9 cells and examine whether the MCP could be expressed in GSM cells. As shown in Figure 1C,D, MCP-specific green fluorescence can be observed in the infected Sf9 cells with the recombinant baculoviruses (Figure 1D), whereas there was no specific fluorescence signal in the normal Sf9 cells (negative control) (Figure 1C). Furthermore, it was shown that MCP was expressed on the surface of the infected Sf9 cells (indicated by arrows). Likewise, MCP-specific red fluorescence can be observed in the GSM cells transduced with the recombinant baculoviruses (indicated by arrows) (Figure 1F), while no specific fluorescence signal was detected in the control (Figure 1E).

### 3.2. Antibody Responses in Chinese Giant Salamanders Immunized with the Recombinant Baculoviruses

**Figure 2:**
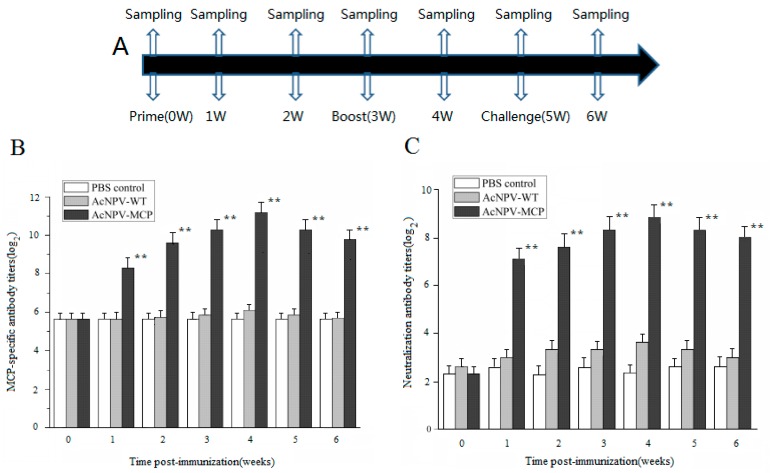
Humoral immune responses induced by AcNPV-MCP. (**A**) Immunization program showing that immunizations were performed in which samples of sera and spleens were collected. (**B**) The changes of MCP-specific serum antibody titers in Chinese giant salamanders immunized with 1 × 10^8^ plaque forming unit (PFU) of AcNPV-MCP (AcNPV-MCP group), wild-type AcNPV (AcNPV-WT group) and 1 mL PBS (PBS control group) respectively. Blood samples were collected from three animals from each group randomly at weeks 0, 1, 2, 3, 4, 5 and 6 after the primary immunization. The endpoint titers were expressed as the reciprocal of the highest sample dilution for which the optical density was equal to or greater than the mean optical density of preimmune sera. Data are presented as mean ± SD (*n* = 3). ** *p* < 0.01. (**C**) The changes of serum neutralization antibody titers in Chinese giant salamanders immunized with 1 × 10^8^ PFU of AcNPV-MCP (AcNPV-MCP group), wild-type AcNPV (AcNPV-WT group) and 1 mL PBS (PBS control group) respectively. Blood samples were collected from three animals from each group randomly at weeks 0, 1, 2, 3, 4, 5 and 6 after the primary immunization. The endpoint titers were expressed as the reciprocal of the highest serum dilution resulting in neutralization of virus activity by 50%. Data are presented as mean ± SD (*n* = 3). ** *p* < 0.01.

The MCP-specific antibody responses induced by the recombinant baculoviruses were determined by ELISA. The specific IgY antibody levels in serum samples from the AcNPV-MCP group increased significantly from seven days after the primary immunization (*p* < 0.05) and reached a peak at 2^11.2±0.57^ at one week after the booster-immunization (Figure 2B). Despite a slight drop, the specific IgY antibody titers in AcNPV-MCP group were still sustained at high levels by week 3 after booster immunization (the last timepoint tested). Conversely, the specific antibody titers in AcNPV-WT and PBS control groups were negligible.

Neutralizing antibody titers were also determined in the same samples. As shown in Figure 2C, the neutralizing antibody titers in sera from immunized animals have a similar changing trend with the specific antibody titers, significantly higher than that of the PBS control group (*p* < 0.05). No neutralizing antibodies were detected (<1:20) in the animals of the AcNPV-WT and PBS control groups.

### 3.3. Cellular Immune Responses in Animals Immunized with the Recombinant Baculovirus of AcNPV-MCP

**Figure 3:**
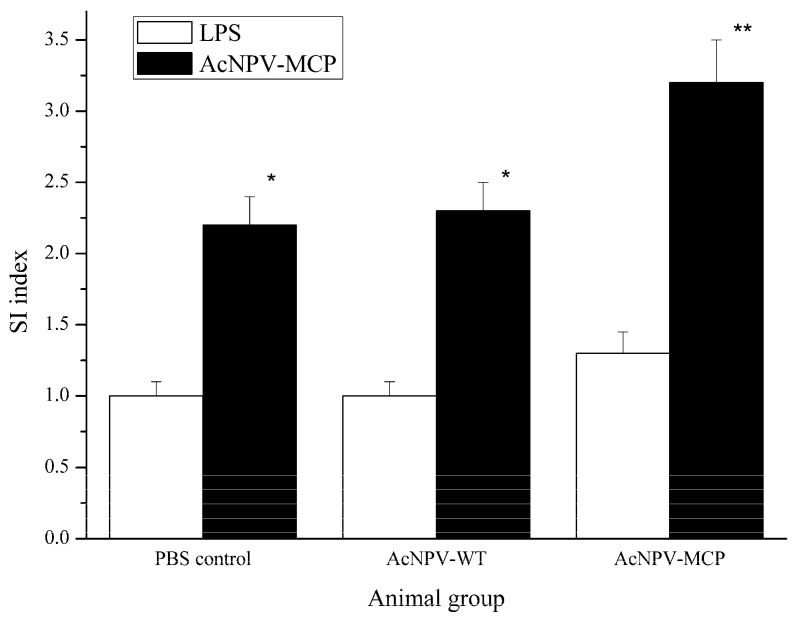
Proliferation of splenocytes response to lipopolysaccharide (LPS) and AcNPV-MCP in Chinese giant salamanders immunized with 1 × 10^8^ PFU of AcNPV-MCP (AcNPV-MCP group), wild-type AcNPV (AcNPV-WT group) and 1 mL PBS (PBS control group) respectively. The splenocytes were sampled at two weeks after the booster immunization. The SI values as described in the Material and Methods section were calculated as an indicator of cell proliferation. Data are presented as mean ± SD (*n* = 3). * *p* < 0.05; ** *p* < 0.01.

**Figure 4:**
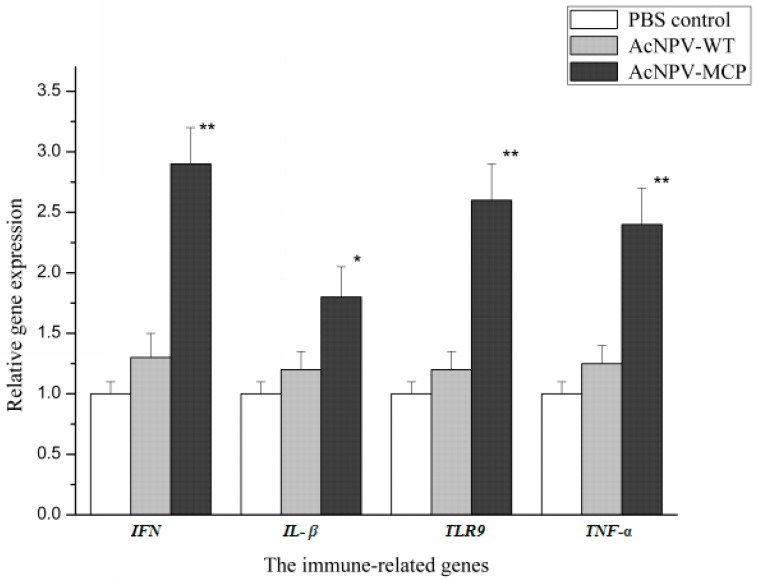
qRT-PCR analysis of the expression of immune-related genes in Chinese giant salamanders immunized with 1 × 10^8^ PFU of AcNPV-MCP (AcNPV-MCP group), wild-type AcNPV (AcNPV-WT group), and 1 mL PBS (PBS control group) respectively. Total RNA was extracted from the spleen tissues at two weeks after the booster immunization used for qRT-PCR. The mRNA level of each gene was normalized to that of *β-actin*. For each gene, the mRNA level of the control group was set as 1. Data are presented as mean ± SD (*n* = 3), * *p* < 0.05; ** *p* < 0.01.

To examine whether the recombinant baculovirus induced antigen-specific cellular immune response, we conducted a splenocyte proliferation assay using CCK-8 method and tested the relative expression levels of the immune genes with quantitative real-time-PCR (qRT-PCR) assays outlined in the section of materials and methods. Figure 3 shows that the splenocytes collected from the AcNPV-MCP group could be significantly induced to proliferate by the recombinant baculovirus compared with those of negative control group (*p* < 0.05). Expression profile of the immune-related genes indicates that significant up-regulation of the expression levels of the four genes in AcNPV-MCP group occurred compared that in PBS control group (*p* < 0.05), while there are only comparable levels in AcNPV-WT group (Figure 4). In addition, highly significant up-regulation of the expression of the three genes including IFN, TNF-α and TLR9 in AcNPV-MCP group can be observed relative to that of those genes in the PBS control group (*p* < 0.01). These results suggest that the recombinant baculovirus could elicit robust cellular immune responses.

### 3.4. Protective Efficacy of the Recombinant Baculovirus

**Figure 5:**
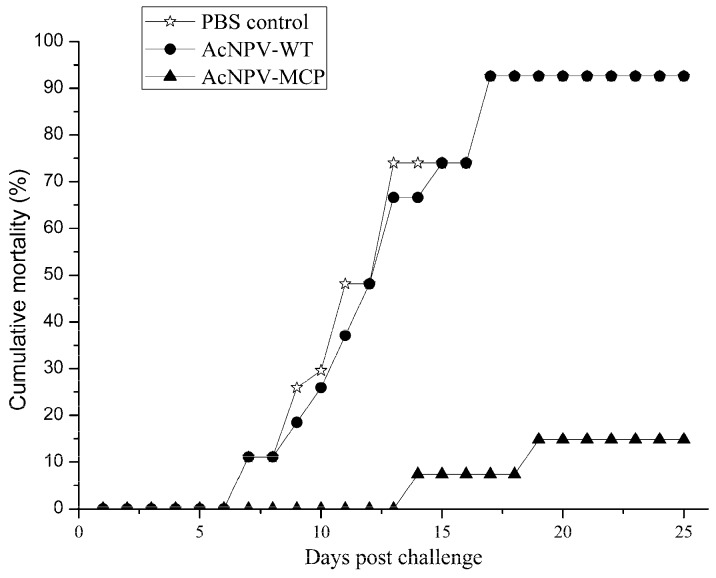
Cumulative mortalities of immunized Chinese giant salamanders upon challenge with Chinese giant salamander iridovirus (CGSIV) at two weeks after the booster immunization. Immunized animals were intraperitoneally challenged with 100 µL of live CGSIV (1.0 × 10^5.5^ TCID_50_/mL). The mortality was recorded daily for up to 25 days post-challenge.

To address whether the recombinant baculovirus confer protective efficacy against CGSIV, we performed a challenge trial in which each animal in all three groups were inoculated ip with lethal dose of CGSIV. As shown in Figure 5, for the AcNPV-WT and PBS control groups, the mortalities were observed from days 7 to 21 after challenge and their cumulative mortalities were 92.6% (25/27); for the AcNPV-MCP group, the mortalities were also observed from days 14 to 21 after challenge, and the cumulative mortalities was 14.8% (4/27). Thus, the RPS of the AcNPV-MCP group was calculated as 84.0% according to the formula presented in materials and methods section.

## 4. Discussion

The MCP comprising 40–45% of the total polypeptides of the ranavirus plays important roles in encapsidation of the virion and in the induction of immune responses [4,27,28], which enables its applications for genetic vaccine candidates against the ranavirus infections [10,29]. In the present study, we have successfully developed a recombinant baculovirus based vaccine expressing the MCP against CGISV infection in Chinese giant salamanders.

To confirm the correct expression of CGSIV MCP by the recombinant baculovirus, Western blot and IIF assays were conducted. The samples of the purified MCP and protein extracts from the recombinant baculovirus-infected Sf9 cells were probed with MCP-mAb showing the similar immunoreactive bands with a molecular weight of about 53 kDa, slightly greater than the predicted size of 50 kDa. This could be attributed to eukaryotic post-translational modifications of the baculovirus expression system [15]. The result of IIF assays indicated that the expressed MCP was expressed on the surface of Sf9 cells infected with the recombinant baculovirus and also in the GSM cells, thus enabling the direct application of the recombinant baculovirus as a vaccine candidate [30].

Antibody responses play very important roles against viral infections. In this study, the specific humoral immune responses to CGSIV MCP were investigated based on ELISA and seroneutralization test. As the data from the two assays showed, significantly higher systemic MCP-specific IgY and accompanied neutralizing antibody responses were detected since week 1 after primary immunization, followed by gradual increase and a peak at week 1 after booster immunization in immunized group with AcMPV-MCP. Similar phenomenon was observed in immunized mice with baculovirus-based vaccine candidate [31,32]. Moreover, the results are also consistent with the previous observations on fish and other amphibians [33,34,35]. Maniero et al. [34] found that frogs (*Xenopus laevis*) inoculated with the type species of *Ranavirus*, frog virus 3 (FV3) up to 15 months after priming produce specific, thymus-dependent anti-FV3 IgY antibodies present in the sera, which are neutralizing in vitro and provide partial passive protection to susceptible larvae. The antibody responses elicited by the recombinant baculovirus vaccination suggest that the specific humoral immunity was triggered by the recombinant baculovirus vaccine and will provide immune protection against CGSIV challenge.

In addition to the humoral responses, immunization with the recombinant baculovirus also efficiently stimulated splenocyte proliferation and induced high expression levels of the immune genes like *INF*, *IL-1β*, *TNF-α*, and *TLR9*. The spleen, an important immune organ in vertebrates, is the site at which antigens from the blood are processed. Therefore, it is not surprising that replication and abundant ranavirus antigen-positive cells were detected in the spleen upon ranavirus infection [36,37]. Thus, in this study, stimulated splenocyte proliferation with the recombinant baculovirus reflects that the immune system was activated and gave an enhanced immune reaction, as similarly shown in a previous study [34] and confirmed by the results of the immune gene expression level.

As critical mediators of host defense mechanisms, type I IFNs (IFN) play very important roles in innate antiviral immunity in vertebrates [38,39]. Grayfer et al. [40] found that *X. laevis* IFN substantially reduced FV3 virus replication and infectious viral burdens in vitro. In a recent in vitro study, Chen et al. [41] found that Chinese giant salamander type I IFN (gsIFN) can reduce the CGSIV MCP synthesis, thus exert a significant antiviral effect on CGSIV replication. It was shown in our study that significant up-regulation level of the IFN gene expression occurred in immunized group with the recombinant baculoviruses, suggesting that the MCP could induce the up-regulation expression of the gsIFN gene and a positive correlation between IFN response and high efficacy of the recombinant baculovirus vaccine. To date, this is the first direct observation that viron-associated viral proteins are the inducer of IFN in aquatic organisms, although it is well known that viral glycoproteins can induce the up-regulation expression of IFN in mammals [42]. Surely, up-regulation IFN-related genes was extensively observed in plasmid DNA vaccinated aquatic organisms [43,44,45]. Ou-yang et al. [45] reported that all of three prepared DNA vaccines show a modest protection against Singapore grouper iridovirus (SGIV), a ranavirus and can up-regulate expression of *Mx* genes in vaccinated grouper (*Epinephelus* pp). The antigens in these three DNA vaccine include SGIV MCP and a viral envelop protein. That is to say that the viral proteins can up-regulate the expression of *Mx* genes, which are usually used as indicator for type I inferno (IFN) system induction. These findings suggest that there exist some unique mechanisms underlying the activation of IFNs by viral proteins in aquatic organisms, which remain to be clarified in future.

However, a well-known and well-accepted notion is that type I IFN response of host after viral infection is initiated through recognition of viral products by host pattern recognition receptors (PRRs) including Toll-like receptors (TLRs) and retinoic acid-inducible gene I (RIG-I)-like receptors’, and then such recognition events trigger distinct signaling pathways and ultimately turn on the transcription of type I IFNs [46]. TLR9, one of many TLRs in host cells, are capable of activating downstream signaling to initiate IFN response. As one of the examined immune-related gene in our study, the expression of TLR9 was significantly up-regulated in AcNPV-MCP group compared to those in the other two treated group. Of course, the up-regulate expression of TLR9 in the AcNPV-WT group was observed compared to that in PBS control group. Previous studies have indicated that baculovirus-based vaccine could induced immune responses in host which are associated with the TLR9-dependent signaling pathway [47]. The results of our study suggest that the activation of TLR9-dependent signaling pathway is further strengthened through immunization with the recombinant baculovirus, which ultimately lead to secretion of IFN in host immune organs to confer protection against viral infection. It is therefore tempting to speculate that the response of IFNs of Chinese giant salamanders is also initiated by the TLR9-dependent signaling pathway and that CGSIV MCP possibly plays some roles in this activating process. However, the underlying mechanism remains elusive and is subjected to investigation further.

As other kinds of cellular responses, inflammatory responses represented by the induction of inflammatory cytokines including TNF-α and IL-1β are critical for viral clearance following ranavirus infection. Morales et al. [48] observed that there were significant increases in the expression of *X. laevis* TNF-α and elevated expression of the IL-1β after the challenge of model types of ranavirus, FV3. De Jesús Andino et al. [33] found that tadpoles of *X. laevis* showed poor and considerably delayed anti-FV3 inflammatory gene responses, in contrast with the robust and quick up-regulation of these inflammatory genes including TNF α and IL-1 β in infected adults, suggesting that the adult have an effective and well-coordinated antiviral immune response. The underlying mechanism is that up-regulation of inflammatory cytokines and then resulted inflammatory migration will be of benefit for simultaneously establishing localized action of innate immune response that ultimately lead to adaptive immunity. In our study, significant up-regulation expression of the two genes TNF-α and IL-1β was observed in the immunized group with the recombinant baculoviruses, demonstrating that the innate immune response and associated inflammatory responses were elicited by the recombinant baculovirus vaccination and confer immune protection against CGSIV challenge. Similar results are manifested in other baculovirus-based vaccine trials [49].

Taken together, as the recombinant baculovirus vaccination effectively elicited both robust humoral and cellular immune responses, the AcNPV-MCP group showed significant immune protection with a RPS of 84% against CGSIV in Chinese giant salamanders compared to the PBS control group in the challenge test.

In conclusion, we have successfully developed a baculovirus-based vaccine candidate against CGSIV infection in Chinese giant salamanders. However, more efforts should be made to further study methods to combat this infection. First, most of present studies focus on the transduction of mammalian cells by the recombinant baculovirus vector, while there are few studies on the factors affecting the efficiency of transduction of the cells of aquatic organisms by baculovirus vectors and better baculovirus vectors fitting for the aquatic organisms. Second, the mechanism underlying the induction of innate immunity in aquatic organisms by the baculovirus-based vaccines remains to be clarified in detail. Although it is reported that wild-type baculovirus can confer complete immune protection against a lethal influenza challenge in intranasally immunized mice [50], this is not the case in our study. Finally, the immunization program in our study needs to be improved via the evaluation of dosage and routes of the immunization with the baculovirus-based vaccine to provide the highest protective immunity against CGSIV infection in Chinese giant salamanders.

## Figures and Tables

**Table 1:** Primers used for PCR analysis.

Gene Name	Nucleotide Sequence (5′→3′)	Restriction Site	Usage	Accession Number
*MCP*	Fw CCGGAATTCATGTCTTCTGTAACTGG Rev CCCAAGCTTTTACAAGATTGGGAATCC	*Eco*R I *Hin*d III	PCR	KF023635
*IFN*	Fw ATTGGCGTGCCTTTTCGTGCTATT Rev GGGAAAGTGTCCACCCATCTGCTC		qRT-PCR	KM267637
*IL-1 β*	FwACCTTCCGGAAGGCAGTGGT Rev TTCTGCCATGGAGGTGACGT		qRT-PCR	KM103345
*TNF-α*	Fw ATGCCAGGACCAATGCTGGA Rev GGCCAGGTGCCTGGTAAGAA		qRT-PCR	KM079078
*TLR9*	Fw GGTGGTTTTGATGCGTTTATTGTATTTC Rev ATTGTGTTGTTCGTGTTTCCTCCAGGTG		qRT-PCR	JN969980
*β-actin*	Fw TGAACCCAAAAGCCAACCGAGAAAAGAT Rev TACGACCAGAGGCATACAGGGACAGGAC		qRT-PCR	HQ822274

*MCP* indicates the gene encoding the major capsid protein of Chinese giant salamander iridovirus; *IFN* indicates the gene encoding type I interferon in Chinese giant salamanders; *IL-1 β* indicates the gene encoding interleukin-1β in Chinese giant salamanders; *TNF-α* indicates the gene encoding tumor necrosis factor alpha in Chinese giant salamanders; *TLR9* indicates the gene encoding toll-like receptor 9 in Chinese giant salamanders; qRT-PCR indicates quantitative real-time-PCR.
